# Supplementary material for: Anthropogenic forcings reverse a simulated multi-century naturally-forced Northern Hemisphere Hadley cell intensification
Source: Nat Commun. 2024 May 11;15:4001. doi: 10.1038/s41467-024-48316-y (PMC11088628; doi:10.1038/s41467-024-48316-y)
Supplement: Supplementary file 1 — Supplementary Information [file 41467_2024_48316_MOESM1_ESM.pdf]

Supplementary Information for ”Anthropogenic forcings reverse a simulated multi-century naturally-forced Northern Hemisphere Hadley cell intensification”

Or Hess[1*] and Rei Chemke[1]

[1]Department of Earth and Planetary Sciences, Weizmann Institute of Science, Rehovot, Israel
[*]Corresponding author: Or Hess; Email: or.hess@weizmann.ac.il

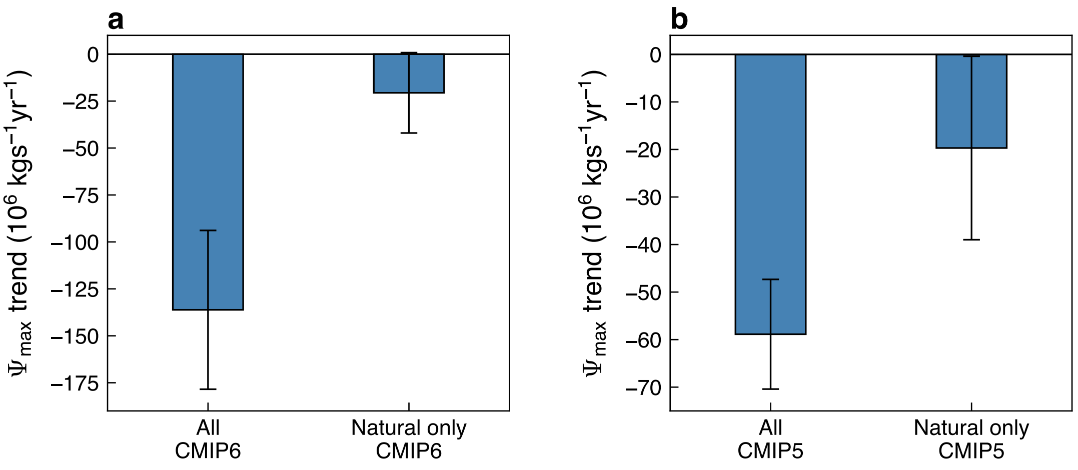

Supplementary Figure 1. The Ψ_{max} trend in **a**, CMIP6 mean between 1970-2014 and in **b**, CMIP5 mean between 1970-2005, under the historical (All) and hist-nat (Natural only) experiments. Error bars show the 95% confidence interval based on a Student's t-distribution.

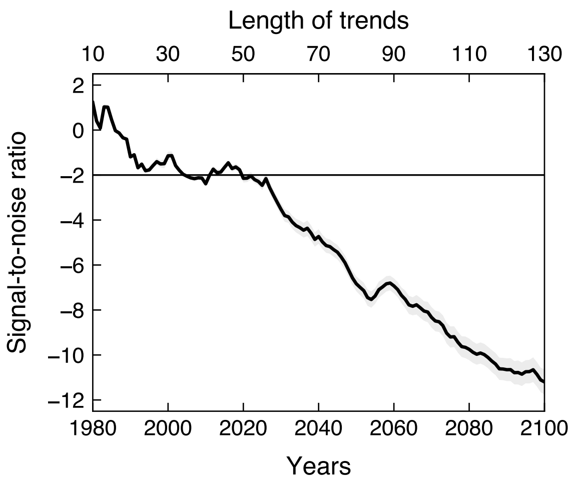

Supplementary Figure 2. Signal-to-noise ratio analysis to the Ψ_{max} trend from 1970 and to each year plotted against the last year of trend in CMIP5 mean (under the historical and the RCP4.5 scenario). Shading shows the s.d. of signal-to-noise ratio values (Methods). The horizontal black line represents a signal-to-noise ratio value of -2.

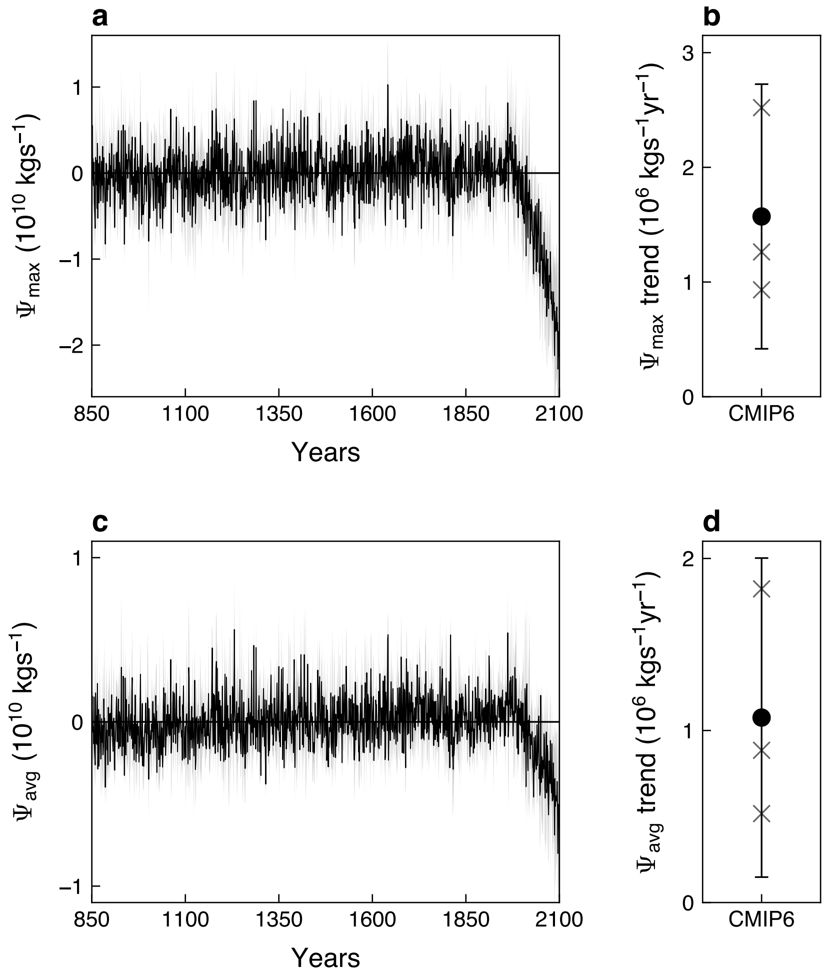

Supplementary Figure 3. **a,c**, Evolution of the Ψ_{max} (**a**) and Ψ_{avg} (**c**), relative to the 1810-1850 period, in CMIP6 mean. Shading shows s.d. across models. **b,d**, The 850-1849 Ψ_{max} (**b**) and Ψ_{avg} (**d**) trends in CMIP6. The black dot shows the mean trend, and the crosses show the individual models' trends. The error bar shows the 95% confidence interval of the mean trend based on a Student's t-distribution.

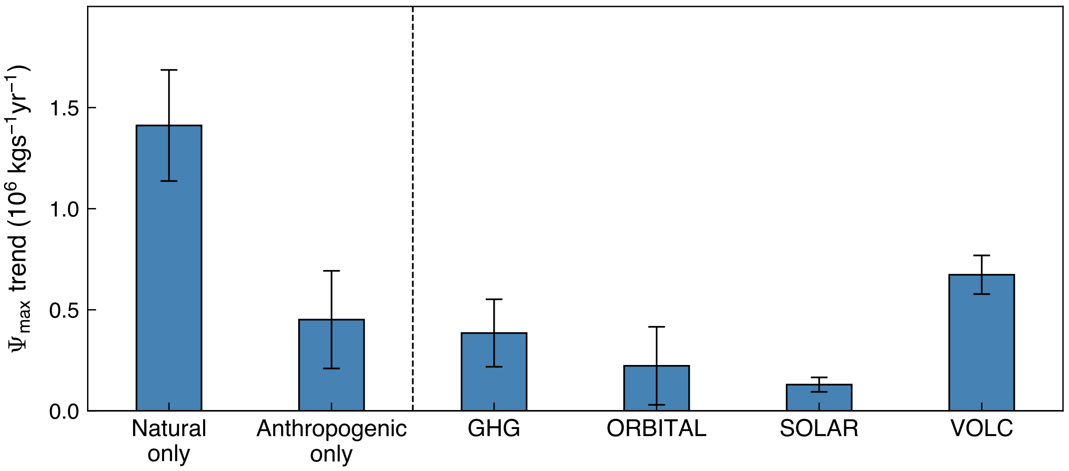

Supplementary Figure 4. The contribution to Ψ_{max} trend in CESM-LME mean between 850-1849 from natural forcings (Natural only) and anthropogenic forcings (Anthropogenic only, i.e., land-use land cover). Right to the dashed line is the decomposition of the contribution of natural forcings from: greenhouse gases (GHG), orbital (ORBITAL), solar (SOLAR), and volcanic (VOLC) changes. Error bars show the 95% confidence interval based on a Student's t-distribution.

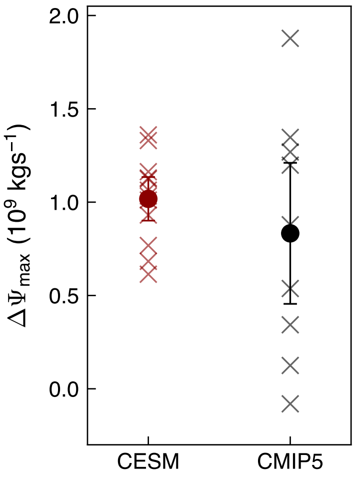

Supplementary Figure 5. The $\Delta\Psi_{max}$ (the difference between the LIA and MCA) in CESM (red) and CMIP5 (black). The red and black dots show the CESM and CMIP5 mean, respectively, and the crosses show the individual members/models. Error bars show the 95% confidence interval of the mean difference based on a Student's t-distribution.

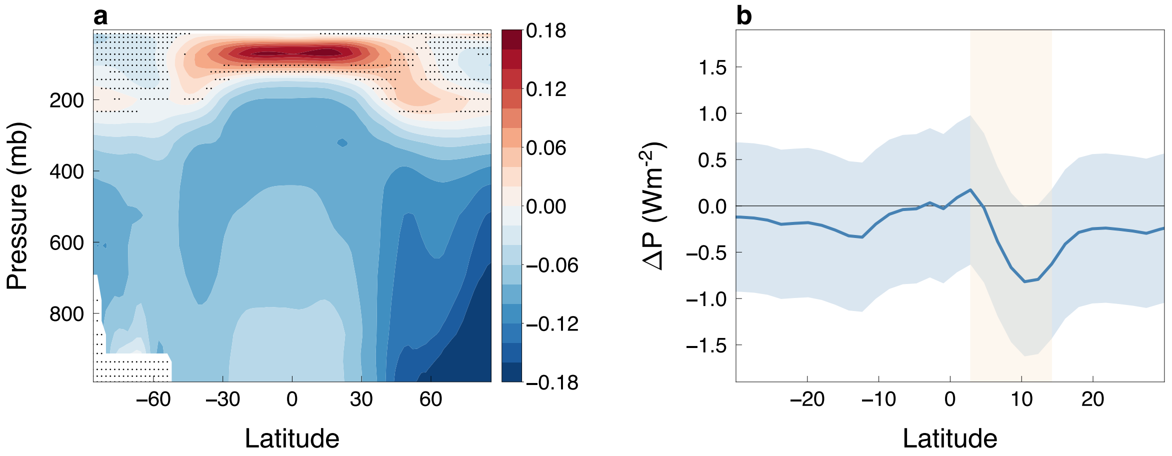

Supplementary Figure 6. The difference between the LIA and MCA of the zonal mean **a**, temperature (K) and **b**, precipitation (ΔP) in CMIP5 mean. The black dots in panel a show where less than two-thirds of the models agree on the sign of change. Blue shading in panel b shows s.d. across the models and orange shading the NH HC ascending branch region.

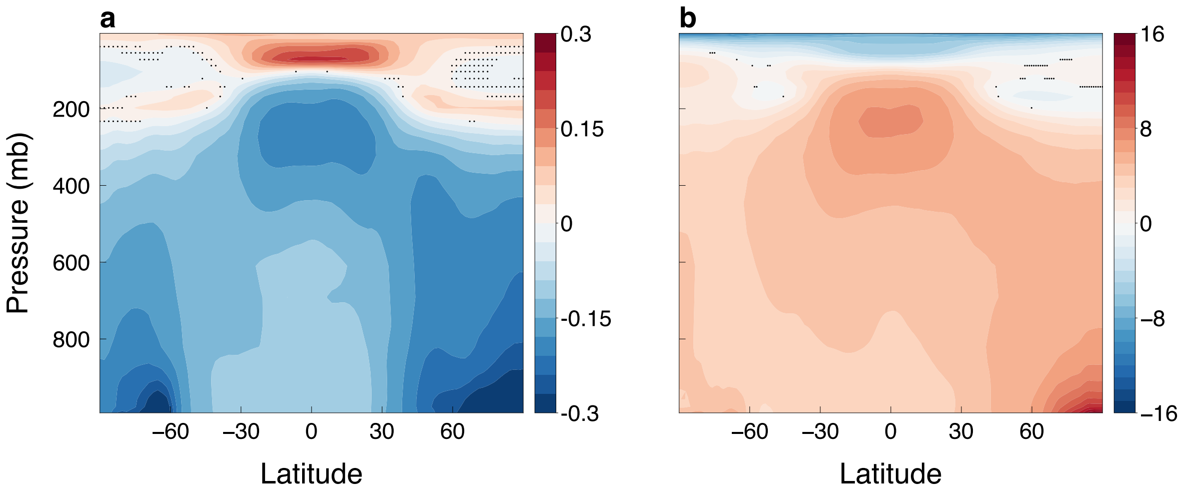

Supplementary Figure 7. The zonal mean temperature difference (K) between **a**, the LIA (1450-1850) and MCA (950-1250) in CESM-LME mean and **b**, the last 20 years of the 21st and 20th centuries in CESM-LE mean. The black dots show where less than two-thirds of the members agree on the sign of change.

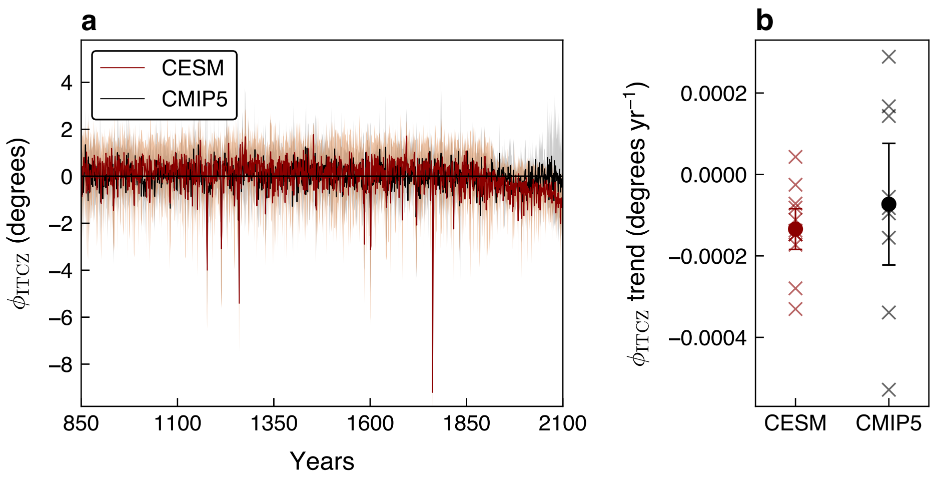

Supplementary Figure 8. **a**, Evolution of the ITCZ position (ϕ_{ITCZ}, defined as the latitude where the 500 mb meridional mass streamfunction, Ψ, is zero), relative to the 1810-1850 period, in CESM mean (red line) and in CMIP5 mean (black line). Shading shows s.d. across members/models. **b**, The 850-1849 ϕ_{ITCZ} trends in CESM (red) and CMIP5 (black). The red and black dots show the CESM and CMIP5 mean trends, respectively, and the crosses show the individual members/models. Error bars show the 95% confidence interval of the mean trend based on a Student's t-distribution.

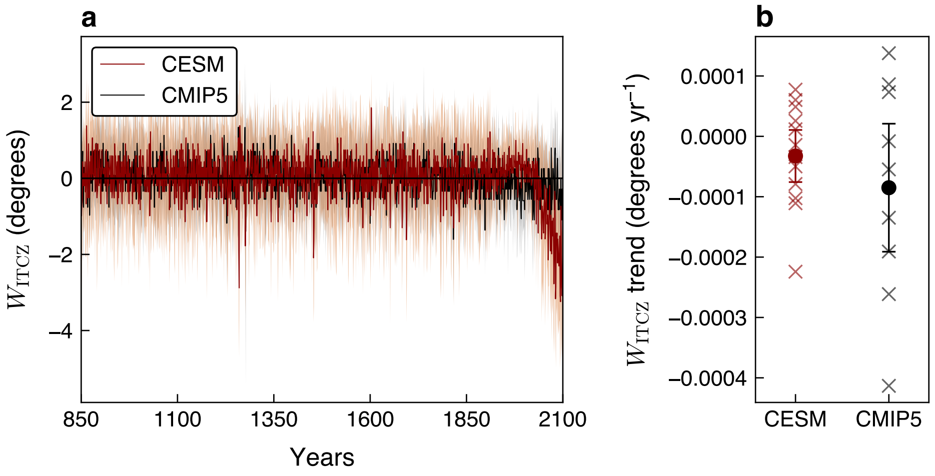

Supplementary Figure 9. **a**, Evolution of the ITCZ width (W_{ITCZ}, defined as the distance in degrees latitude between the ITCZ edges, which are the latitudes closest to the equator at which the meridional derivative of the 500 mb meridional mass streamfunction, $\frac{\partial\Psi}{\partial\phi}$, is zero), relative to the 1810-1850 period, in CESM mean (red line) and CMIP5 mean (black line). Shading shows s.d. across members/models. **b**, The 850-1849 W_{ITCZ} trends in CESM (red) and CMIP5 (black). The red and black dots show the CESM and CMIP5 mean trends, respectively, and the crosses show the individual members/models. Error bars show the 95% confidence interval of the mean trend based on a Student's t-distribution.

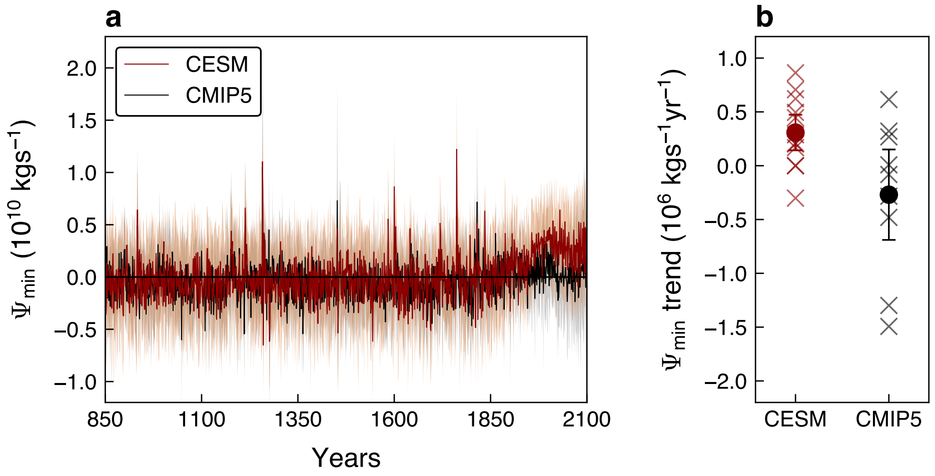

Supplementary Figure 10. a, Evolution of the SH HC strength (Ψ_{min}, defined as the minimum value, at 500 mb, of the meridional mass streamfunction), relative to the 1810-1850 period, in CESM mean (red line) and in CMIP5 mean (black line). Shading shows s.d. across members/models. **b**, The 850-1849 Ψ_{min} trends in CESM (red) and CMIP5 (black). The red and black dots show the CESM and CMIP5 mean trends, respectively, and the crosses show the individual members/models. Error bars show the 95% confidence interval of the mean trend based on a Student's t-distribution.

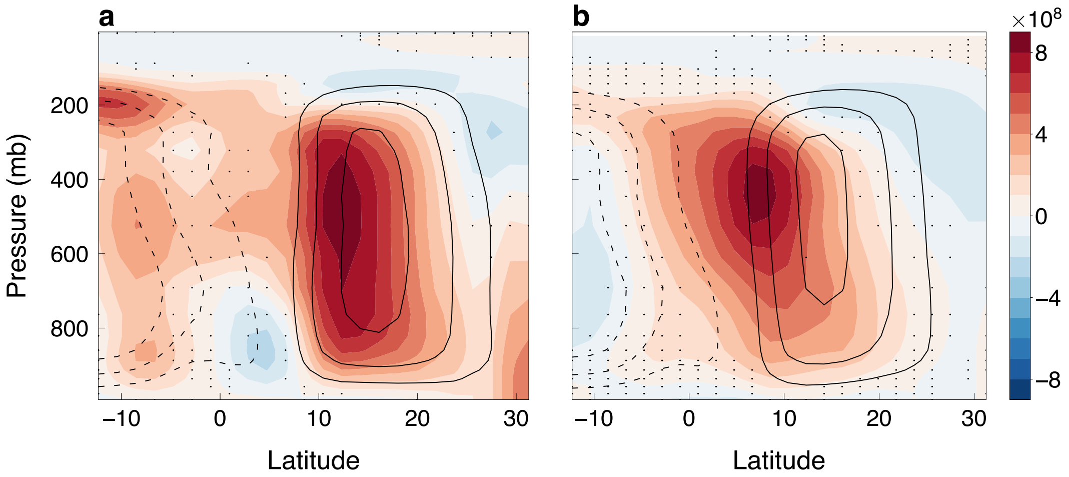

Supplementary Figure 11. The difference between the LIA and the MCA periods of the zonally mean meridional mass streamfunction ($\Delta\Psi$, kgs^{-1}) in **a**, CESM-LME mean and **b**, CMIP5 mean. Black contours show the streamfunction averaged over the MCA period (dashed lines represent negative values) in intervals of 2.5×10^{10} kgs^{-1}, with a maximum absolute value of 7.5×10^{10} kgs^{-1}. Black dots show where less than two-thirds of the members/models agree on the sign of change.

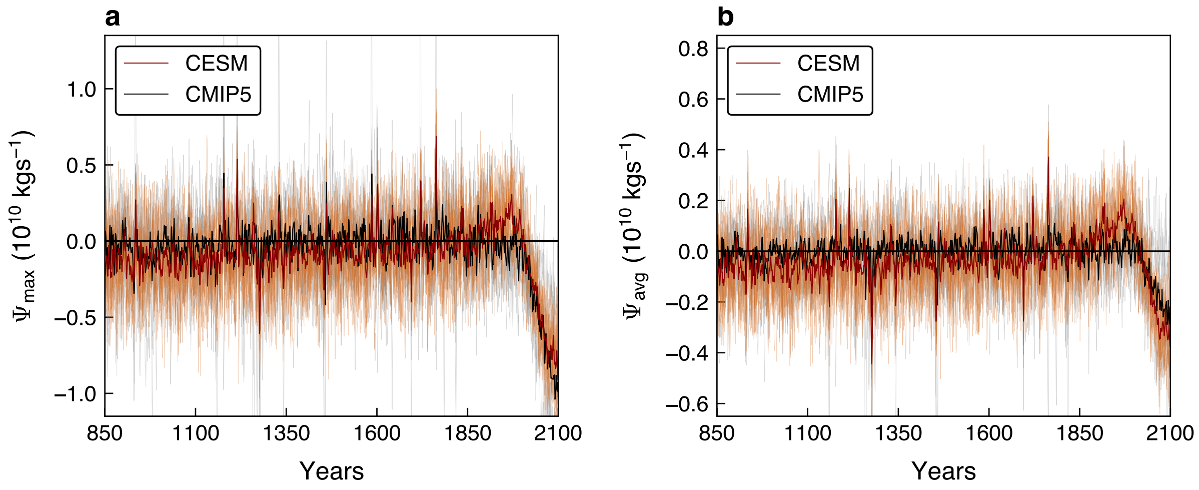

Supplementary Figure 12. Evolution of **a**, Ψ_{max} and **b**, Ψ_{avg}, relative to the 1810-1850 period, in CESM mean (red line) and in CMIP5 mean (black line). Thin lines show the evolution of individual members/models. The evolution has been smoothed with a 3-year running mean for plotting purposes.

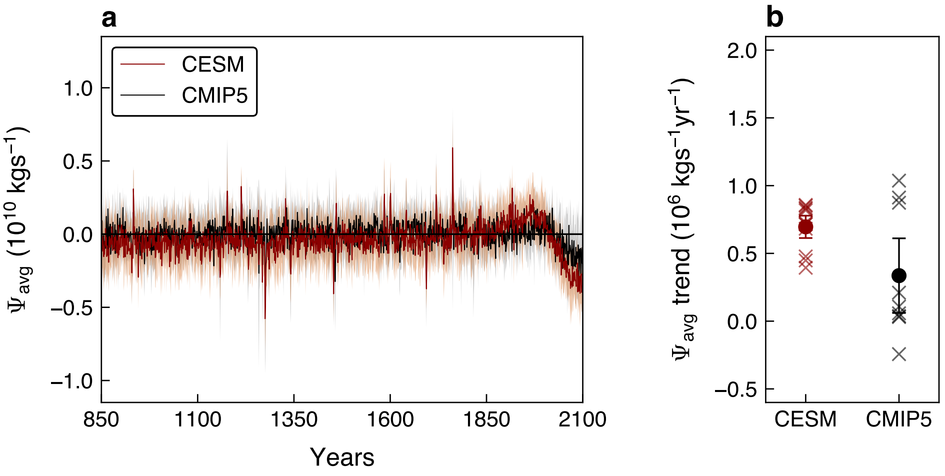

Supplementary Figure 13. **a**, Evolution of Ψ_{avg}, relative to the 1810-1850 period, in CESM mean (red line) and in CMIP5 mean (black line). Shading shows s.d. across members/models. **b**, The 850-1849 Ψ_{avg} trends in CESM (red) and CMIP5 (black). The red and black dots show the CESM and CMIP5 mean trends, respectively, and the crosses show the individual members'/models' trends. Error bars show the 95% confidence interval of the mean trend based on a Student's t-distribution.

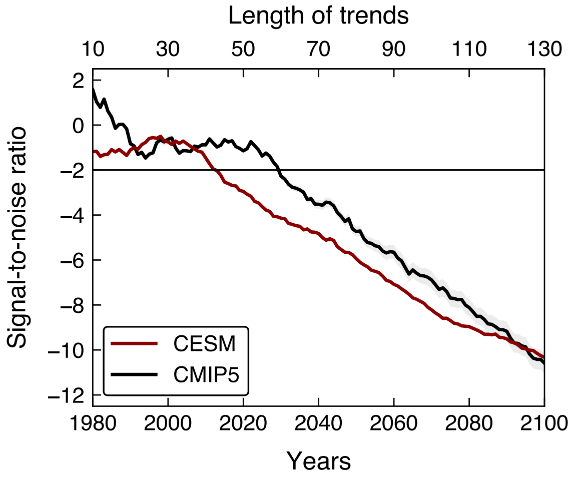

Supplementary Figure 14. Signal-to-noise ratio analysis to the Ψ_{avg} trend from 1970 and to each year plotted against the last year of trend in CESM mean (red line) and CMIP5 mean (black line). Shading shows the s.d. of signal-to-noise ratio values (Methods). The horizontal black line represents a signal-to-noise ratio value of -2.

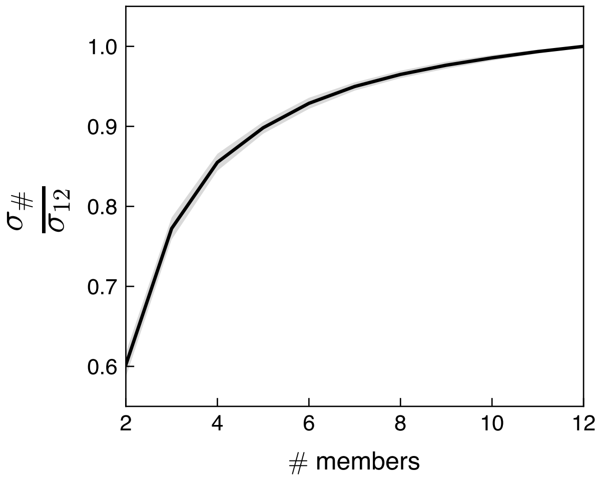

Supplementary Figure 15. Fractional change in s.d. (σ) of the Ψ_{max} across different numbers of CESM-LME ensemble members, relative to the s.d. calculated using all 12 CESM-LME members. The s.d. is calculated at each year and averaged over the 850–1849 period and over all combinations of numbers of ensemble members (or up to 1000 random combinations). Shading shows s.d. across the different combinations of the number of ensemble members.

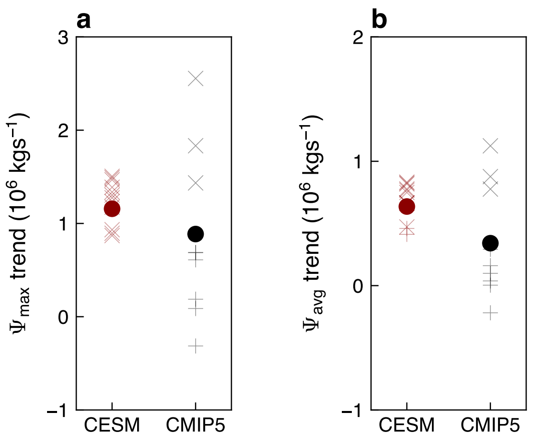

Supplementary Figure 16. The 850-1849 **a**, Ψ_{max} and **b**, Ψ_{avg} trends in CESM (red) and CMIP5 (black) based on the Mann-Kendall test (Methods). The red and black dots show the CESM and CMIP5 mean trends, respectively, and the crosses (pluses) show the individual models' trends with p-values less (more) than 0.05. Note that the mean trend for both ensembles is significant, with p-values less than 0.05.

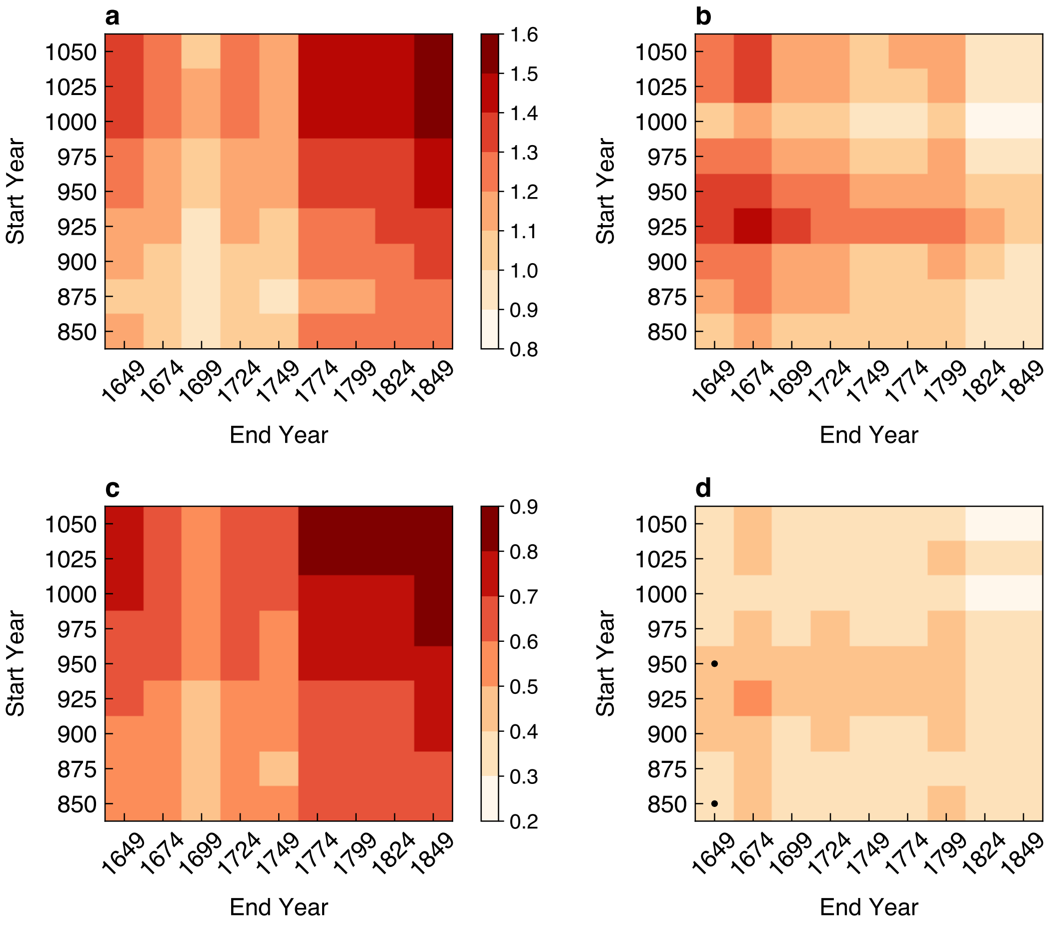

Supplementary Figure 17. Trends ($kgs^{-1}yr^{-1}$) in Ψ_{max} (**a,b**) and Ψ_{avg} (**c,d**) in the mean of CESM (**a,c**) and CMIP5 (**b,d**). Black dots show where less than two-thirds of the members/models agree on the sign of the trend.
